# Temporal analysis of water chemistry and smallmouth bass (*Micropterus dolomieu*) health at two sites with divergent land use in the Susquehanna River watershed, Pennsylvania, USA

**DOI:** 10.1007/s10661-024-13049-4

**Published:** 2024-09-11

**Authors:** Heather L. Walsh, Geoffrey D. Smith, Megan K. Schall, Stephanie E. Gordon, Vicki S. Blazer

**Affiliations:** 1 Eastern Ecological Science Center, Leetown Research Laboratory, U.S. Geological Survey, 11649 Leetown Rd, Kearneysville, WV 25430 USA; 2 Pennsylvania Fish and Boat Commission, Division of Fisheries Management, 595 E. Rolling Ridge Drive, Bellefonte, PA 16823 USA; 3 Biological Services, Pennsylvania State University–Hazleton, 76 University Drive, Hazleton, PA 18202 USA

**Keywords:** Fish health, Contaminants, Susquehanna River watershed, Smallmouth bass, Agriculture, Indicator species

## Abstract

Monitoring wild fish health and exposure effects in impacted rivers and streams with differing land use has become a valuable research tool. Smallmouth bass (*Micropterus dolomieu*) are a sensitive, indicator species that exhibit signs of immunosuppression and endocrine disruption in response to water quality changes and contaminant exposure. To determine the impact of agriculture and development on smallmouth bass health, two sites (a developed/agriculture site and a forested site) in the Susquehanna River watershed, Pennsylvania were selected where bass and water chemistry were sampled from 2015 to 2019. Smallmouth bass were sampled for histopathology to assess parasite and macrophage aggregate density in the liver and spleen, condition factor (Ktl), hepatic gene transcript abundance, hepatosomatic index (HSI), and a health assessment index (HAI). Land use at the developed/agriculture site included greater pesticide application rates and phytoestrogen crop cover and more detections and higher concentrations of pesticides, wastewater-associated contaminants, hormones, phytoestrogens, and mycotoxins than at the forested site. Additionally, at the developed/agriculture site, indicators of molecular changes, including oxidative stress, immune/inflammation, and lipid metabolism-related hepatic gene transcripts, were associated with more contaminants and land use variables. At both sites, there were multiple associations of contaminants with liver and/or spleen macrophage aggregate density, indicating that changes at the molecular level seemed to be a better indicator of exposures unique to each site. The findings illustrate the importance of timing for land management practices, the complex mixtures aquatic animals are exposed to, and the temporal changes in contaminant concentration. Agricultural practices that affect hepatic gene transcripts associated with immune function and disease resistance were demonstrated which could negatively affect smallmouth bass populations.

**Supplementary Information:**

The online version contains supplementary material available at 10.1007/s10661-024-13049-4.

## Introduction

Over the past two decades in the Susquehanna River watershed Pennsylvania (PA), smallmouth bass (SMB; *Micropterus dolomieu*) health, juvenile recruitment, and population structure (a shift to older and larger fish) have been impacted by complex interactions of multiple factors such as changes in land use (agriculture, mining, and development), water quality (Shull & Pulket, 2015; Smith et al., 2015), and pathogens and disease (Starliper et al., 2013; Walsh et al., 2018). A high occurrence of skin lesions and evidence of contaminant accumulation in the tissues of young-of-the-year (YOY) SMB has been observed (Walsh et al., 2018), while adult male SMB have shown a high rate and severity of intersex (testicular oocytes) associated with agricultural land use (Blazer et al., 2014). Although multiple factors potentially related to these health issues have been identified, there has not been one consistently associated with observed effects. More recently, however, the population size structure has become more stable across size classes (Schall et al., 2020). Additionally, disease occurrence in juvenile SMB was shown to decrease from 2013 to 2016; however, an increase was observed again in 2017 demonstrating seasonal and annual variations in disease (Schall et al., 2020).

Despite years of research, there is still much to be learned regarding the risk factors associated with SMB morbidity and disease in this system. Due to their endocrine disruption sensitivity (Blazer et al., 2012, 2014) and tendency to bioaccumulate contaminants (Schmitt et al., 2011; Walsh et al., 2018), SMB are an important indicator species in environmental monitoring studies. New research at the molecular level may provide a more comprehensive understanding of exposure effects, including the capability to identify genes associated with exposure and accumulation of specific contaminants, including pesticides (Christiansen et al., 2014). Inclusion of these analyses in fish health assessments can help explain how toxicants and land use variables are associated with health effects at the molecular (gene expression), cellular (histopathology), and organismal levels and provide a foundation for risk assessment and regulatory decision-making at the population level (Villeneuve et al., 2014). While it is meaningful to identify relationships between individual contaminants and health measures, wild fishes are exposed to complex contaminant mixtures (consisting of tens to thousands of chemicals; Feron & Groton, 2002) which are difficult to analyze yet are likely to have a greater health impact than individual contaminants alone (Hamilton et al., 2016; Orton et al., 2014). Oftentimes, these mixtures (parent and transformation products/metabolites) vary temporally due to changes in climate (flow, temperature), water quality (pH, conductivity) (Fisher, 1991; Kim et al., 2010), and organics (Carpio et al., 2021). Changes in concentrations can have varied biological effects (Perkins et al., 2019), particularly when exposures occur at sensitive life stages (Mohammed, 2013). Contaminants, such as phytoestrogens and herbicides (including atrazine), have been shown to induce estrogenic-endocrine disruption in fish (Stevenson et al., 2011; Suzara & Ingraham, 2008), resulting in changes such as a higher female-to-male ratio, testicular oocytes, vitellogenin production, and altered reproductive effects. In addition to surface water contaminants, chemicals that bioaccumulate or bioconcentrate, such as mercury (Willacker et al., 2020) and per- and polyfluoroalkyl substances (Blazer et al., 2021), have been detected in SMB within the Chesapeake Bay watershed.

In 2013, the U.S. Geological Survey (USGS) Environmental Health Program initiated long-term monitoring of sites impacted by a range of agricultural uses in the Chesapeake Bay watershed. One goal of these assessments was to integrate land-use, water chemistry, and SMB health indicators at sites impacted by agriculture and development over multiple years and seasons. As part of this study, fish health assessments of adult SMB (Blazer et al., 2018) were conducted at two sites within the Susquehanna River watershed: one impacted by agriculture/development and one with primarily forested land cover for comparison. The goal of the current study was to perform a long-term assessment of spatio-temporal changes, including land use and water chemistry, associated with multiple SMB biological endpoints at the molecular (hepatic gene transcripts), cellular (parasite and macrophage aggregate densities), tissue (hepatosomatic index; HSI), and individual (health assessment index; HAI) levels.

## Materials and methods

### Land use and surface water analyses

**Fig. 1:**
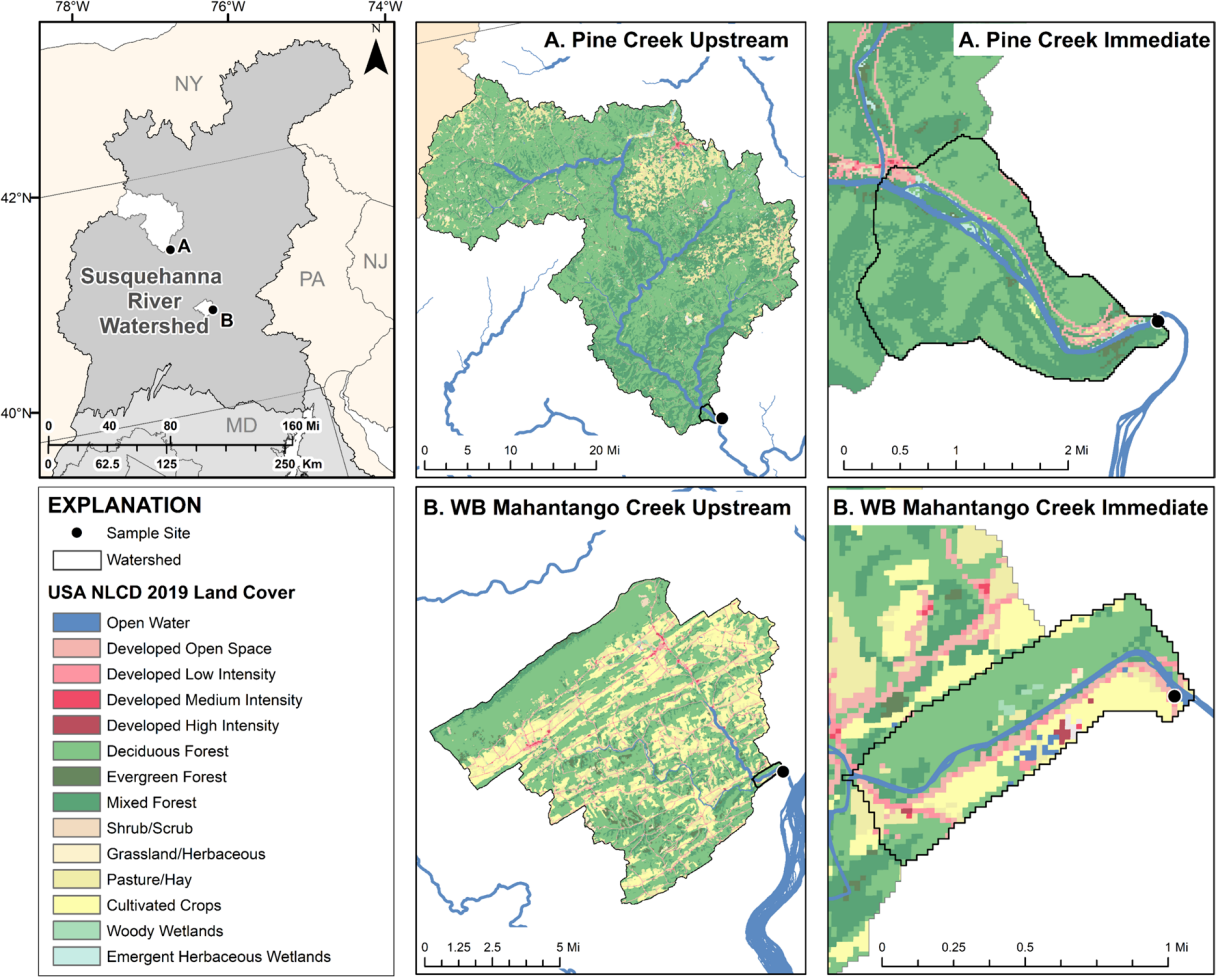
Land use (Dewitz and U.S. Geological Survey, 2021) in the upstream (**A**) and immediate (**B**) catchments surrounding the smallmouth bass sampling sites at Pine Creek and West Branch Mahantango Creek (WBM) located in the Susquehanna River watershed

Two sites from the Susquehanna River watershed were selected based on land use (Fig. 1). The first was located along Pine Creek (Pine Creek at Ramsey, 41.28296, − 77.3215) in the West Branch Susquehanna River watershed, hereafter referred to as Pine. The headwaters start in Potter County and flow southeast for 140.33 km where the creek empties into the West Branch Susquehanna River. The primary land cover around Pine Creek is forest; however, it includes some agriculture, and portions are used for coal mining (associated with acid mine drainage; Pennsylvania Department of Environmental Protection, 2008) and natural gas drilling. The second site, approximately 85 km southeast of the Pine Creek site, was located along the West Branch Mahantango Creek (40.6471, − 76.9656), close to the confluence with Mahantango Creek and hereafter referred to as WBM. The West Branch Mahantango Creek (a tributary of Mahantango Creek) is over 32 km in length, and over 17 km have been listed on the 303(d) list of impaired streams due to sedimentation associated with agriculture (Pennsylvania Department of Environmental Protection, 2008).

For the current study, the immediate and upstream catchments at each site were delineated using the medium-resolution National Hydrography Dataset (Version 2.1, EPA and U.S. Environmental Protection Agency & the U.S. Geological Survey, 2012; Walsh et al., 2022). Land use and landcover data including landcover data from the 2016 and 2019 National Landcover Database (Dewitz & U.S. Geological Survey, 2021; https://www.mrlc.gov/data), number of municipal and industrial wastewater facilities (National Pollutant Discharge Elimination System (NPDES) facilities), percent coverage of high phytoestrogen crop cover (alfalfa, barley, clover/wildflowers, flaxseed, grapes, oats, peanuts, rye, soybeans, hops, and wheat, hereafter referred to as phytoestrogen crop cover), estimated pesticide applications (high pesticide estimates, see Wieben, 2021), nutrients from biosolid applications, and concentrated animal lot operations (CAFOs; Gordon et al., 2017) were summarized at both scales, when applicable (Gordon, 2024).

Similarly, surface water contaminant analysis was conducted as described by Walsh et al. (2022) and Smalling et al. (2021). In brief, surface water was collected in 2015–2019 bi-monthly and during high flow events in the spring (April–June) and once a month for the remainder of the year (July–March). Samples were extracted and analyzed for approximately 300 organic contaminants, including 105 current-use pesticides (Hladik et al., 2008), 107 pharmaceuticals (Furlong et al., 2014), 20 hormones and sterols (Foreman et al., 2012), 52 wastewater indicators (Zaugg et al., 2007), and seven phytoestrogens and eight mycotoxins (Yost et al., 2013). The complete dataset, including methods and reporting limits, can be found in Williams et al. (2019).

Total estrogenicity (E2Eq) was quantified from water samples with a bioluminescent yeast-based estrogen screen (BLYES) and reported relative to 17β-estradiol equivalents as described in Ciparis et al. (2012). The assay indicates the presence of compounds that can bind to the estrogen receptors but does not differentiate between agonists and antagonists (Sanseverino et al., 2005).

Daily mean discharge (cubic feet per second, cfs) was collected from the U.S. Geological Survey National Water Information System’s real-time stream gage at Pine Creek in Waterville (USGS station no. 01549700; U.S. Geological Survey (2020). For WBM, discharge was based on a regression analysis from field-collected data (2016–2017) and from data collected at the nearby Penn’s Creek USGS gage station (USGS station no. 01555000) as described in McClure et al. (2020).

### Smallmouth bass sampling

Approximately 20 adult smallmouth bass (≥ 200 mm) were collected during every sampling effort by boat or tow barge electrofishing in the spring of 2015–2019 and in the fall of 2016–2018. Bass were humanely euthanized with a lethal dose (350 mg/L) of tricaine-methanesulfonate (MS-222, Argent Finquel, Redmond, Washington) according to the U.S. Geological Survey Eastern Ecological Science Center’s Institutional Animal Care and Use Committee protocol (IACUC # 2020–06). All SMB were examined for external abnormalities, weighed to the nearest gram (gm), and measured for total length to the nearest millimeter (mm), and sagittal otoliths were removed for aging (Blazer et al., 2014). An abdominal slit was cut from the vent to the operculum, and the spleen and liver were removed and weighed to the nearest 0.01 gm, and pieces were placed in Z-Fix® (Anatech Ltd., Battle Creek, Michigan) for histological preservation. Small pieces of liver were placed in RNALater® (Thermo Fisher Scientific) according to the manufacturer’s protocol for downstream RNA extraction and analysis and kept at 4 °C for 24 h prior to freezing at − 20 °C until extractions were completed. The hepatosomatic index (HSI) was calculated as (liver weight / (weight − gonad weight)) × 100, and a modified condition factor (Ktl) was calculated as ((weight − gonad weight) / (total length^3^) × 100,000).

### Laboratory analyses

The liver and spleen were fixed in Z-Fix® for ≥ 48 h prior to histological processing. Tissues were embedded in paraffin, sectioned at 5 µm, and stained with hematoxylin and eosin (Luna, 1992). Up to three cross-sections of the liver and one cross-section of the spleen were examined, and parasite and macrophage aggregate (MA) density was determined. Tissues were observed with a Nikon Labophot-2 brightfield microscope (Nikon, Minato City, Tokyo, Japan) with Nikon PlanApo 4 × (for parasite density) and 10 × (for MA density) objectives. Parasite density was calculated as (# of parasites counted in 3 fields of view (FOV) / (FOV area) × 3), and MA density was calculated as (# of macrophage aggregates counted in 5 FOV / (FOV area) × 5). The FOV radius was calculated as ((field number / objective magnification × auxiliary lens magnification) / 2), and the FOV area was calculated as (π × (FOV radius)^2^). In instances where there was not enough tissue to view three or five cross-sections, the equation was adjusted. For example, some pieces of spleen were too small to view in three or five FOV, and only one or two FOV could be observed for parasite density or two to four for MA density and was considered the total number of FOV. Additionally, parasites or MA that were partially outside a FOV were not counted. Parasites were identified at the phylum level (Platyhelminthes) based on characteristics provided by Hoffman (1999).

A modified health assessment index (HAI) was determined for each fish as described in Blazer et al. (2023). It included numerical values based on severity/damage assigned to observed abnormalities of the fins/body surface, gills, eyes, liver, spleen, anterior and posterior kidney, swimbladder, and other variables (gonad and mesentery). The HAI consisted of summed values of abnormalities observed in each organ with higher values indicating a greater number of abnormalities and severity/damage.

Liver preserved in RNALater® was thawed on ice, and 15–25 mg of tissue was extracted for total RNA with the E.Z.N.A. Total RNA Kit I (Omega Bio-Tek, Norcross, Georgia). DNA contamination was removed with a DNase treatment step (RNase-free DNase set I, Omega Bio-Tek), and samples were eluted with 50 µL nuclease-free water. An RNA BR Assay Kit (Agilent, Santa Clara, California) was used to quantify purified RNA on a Qubit 4 Fluorometer. Following extraction, the CodeSet previously published in Hahn et al. (2016) was used for hepatic transcript abundance analysis with 50 ng of RNA/sample on the Nanostring nCounter® SPRINT (Nanostring Technologies, Inc., Seattle, Washington) and included gene transcripts associated with stress (five genes), oxidative stress (five genes), cell proliferation (five genes), contaminant and lipid metabolism (seven genes), thyroid function (three genes), insulin/pancreas function (three genes), and immune/inflammation (nine genes; Table 1).

**Table 1:** Hepatic gene transcripts included in the Nanostring nCounter® CodeSet

Transcript name	Transcript symbol	Related function
40S Ribosomal Protein S12	*40SrpS12*	Housekeeping
Elongation Factor 1A	*ef1α*	Housekeeping
Eukaryotic Translation Initiation Factor 3D	*etif3d*	Housekeeping
Ribosomal Protein L8	*rpl8*	Housekeeping
Aryl Hydrocarbon Receptor	*ahr*	Contaminant metabolism
CYP1A	*cyp1*α	Contaminant metabolism
CYP3A	*cyp3*α	Contaminant metabolism
Metallothionein	*mt*	Contaminant metabolism
Epoxide Hydrolase 1	*eh1*	Contaminant metabolism
Glucokinase	*glk*	Insulin/pancreas
Insulin-like Growth Factor 1	*igf1*	Insulin/pancreas
Phosphoenolpyruvate Carboxykinase	*pepck*	Insulin/pancreas
Thyroid Hormone Receptor Beta	*thrβ*	Thyroid
Type I Deiodinase	*dio1*	Thyroid
Type II Deiodinase	*dio2*	Thyroid
Glucocorticoid Receptor	*gr*	Stress
Warm Temperature Acclimation Protein 65	*wap65*	Stress
Heat Shock Protein 70	*hsp70*	Stress
Heat Shock Protein 71	*hsp71*	Stress
Heat Shock Protein 90b	*hsp90b*	Stress
Catalase	*cat*	Oxidative stress
Glutathione Peroxidase	*gp*	Oxidative stress
Glutathione Reductase	*gsr*	Oxidative stress
Glutathione S-Transferase Theta-1	*gst*	Oxidative stress
Superoxide Dismutase	*sod*	Oxidative stress
Hepcidin 1	*hep1*	Immune/inflammation
Hepcidin 2	*hep2*	Immune/inflammation
Transferrin	*tf*	Immune/inflammation
Transforming Growth Factor Beta	*tgfβ*	Immune/inflammation
C-reactive Protein-like	*crpl*	Immune/inflammation
Complement Component 3	*c3*	Immune/inflammation
Arginase	*arg*	Immune/inflammation
Heme Oxygenase 1b	*ho1b*	Immune/inflammation
Branched-chain aminotransferase	*bcat*	Immune/inflammation
Apolipoprotein A1	*apa1*	Lipid metabolism
Peroxisome Proliferator-Activated Receptor	*ppar*	Lipid metabolism
Proliferating Cell Nuclear Antigen	*pcna*	Cell proliferation
Epidermal Growth Factor Receptor	*egfr*	Cell proliferation
Tata Box Binding Protein	*tbp*	Cell proliferation
B-cell Lymphoma 2	*bcl2*	Cell proliferation
Hypoxanthine Phosphoribosyltransferase 1	*hprt1*	Cell proliferation

Included in the CodeSet were internal positive and negative controls and housekeeping transcripts for normalization which was conducted in nSolver 4.0 (Nansotring Technologies, Inc.). The limit of detection (LOD) was calculated separately for each site as the (mean + (2 × standard deviation)) and was 50 counts for Pine and 41 counts for WBM. The liver was not taken in RNALater® in spring 2016 at WBM; therefore, data from spring 2016 at Pine was not included in any transcript abundance analyses.

### Statistical analyses

All statistical analyses were conducted in R version 4.1.3 (R Core Team, 2023).

A nonparametric, Kruskal–Wallis one-way ANOVA was used to analyze differences in biological variables between years, sexes, and sites (including age, weight, total length, Ktl, HSI, HAI, and liver and spleen parasite and MA density). This type of analysis was used since the data was nonparametric and transformations for normality were unsuccessful; thus, the assumptions for a two-way ANOVA could not be met. A Kruskal–Wallis test was also used to identify gene transcripts that were the same between seasons which were then used in subsequent correlation analyses with land use variables at each site and a PCA to identify relationships between the variables. This method allowed the use of fall data (which only had three observations; 2016, 2017, 2018) so the annual average of the biological variables or gene transcripts was used. For these analyses, the sexes were kept separate since many of the transcripts typically vary by sex.

The “rcorr.adj” function in the package “RcmdrMisc,” was used to produce a Spearman’s rank correlation matrix with a Holm’s correction to identify associations between contaminants at each site with the mean of the biological variables (listed above) and gene transcript abundance. Since biological variables have different response times (i.e., genes respond in hours to days, and HSI, HAI, and parasites and MA densities respond in weeks to months), differences in mean chemical contaminants were used based on these time frames. For example, mean contaminant concentrations sampled 1 week prior to fish collections were used to understand associations with hepatic transcript abundance, and mean contaminant concentrations sampled in March to within 1 week prior to when fish were sampled were used to understand associations with Ktl, HSI, HAI, and parasite and MA density. Since several of the biological variables change by season, only spring was used in these analyses due to not enough fall observations.

An adjusted Spearman’s rank correlation analysis was also used to identify associations between land use variables and average annual contaminant concentration at each site. For this analysis, only contaminants that were detected > 4 times in a given year for > 4 years were included to meet the requirements of the analysis.

Lastly, a principal component analysis (PCA) was used to analyze the variance in gene transcript abundance at each site (with both seasons and all years included). Principal components (PC) that explained ≥ 80% of the variance in the data were included in a multiple linear regression model (mlr) to identify relationships with age, length, weight, Ktl, HSI, HAI, and MA and parasite density. From each PC that was identified as significant, gene transcripts with factor loadings ≥  ± 0.3 were analyzed in subsequent mlr models which also included season, year, and age as fixed effects. If multiple models were identified for each variable, the model with the largest adjusted *R*^2^ was selected. For all statistical analyses, results were considered significant with a *p*-value < 0.05.

## Results

### Land use summaries

The areas of the immediate catchment of Pine and WBM were 8.488 km^2^ and 1.214 km^2^, while the areas of the upstream catchment were 2436.808 km^2^ and 218.489 km^2^, respectively (Gordon 2024). Although the catchment size was smaller, WBM had a greater percentage of developed and agricultural (pasture/crop) land cover, while Pine had more forested land cover (Fig. 1, Table 2).

**Table 2:** Percentage (%) and % change over time of developed (open, low, medium, high), forested (deciduous, evergreen, mixed), and agricultural (pasture/hay, cultivated crops) land cover, and number (#) of septic systems and # of heads of cow, swine, and poultry in concentrated animal feedlot operations (CAFOs) in the upstream and immediate catchments of West Branch Mahantango Creek (WBM) and Pine Creek (Pine)

Land use	NLCD year	WBM (%)	% Change over time	Pine (%)	% Change over time
Developed (immediate)	2016	10.01	+ 1.63	4.18	+ 0.54
	2019	11.64		4.72	
Forest (immediate)	2016	66.05	− 4.37	88.24	− 1.33
	2019	61.68		86.91	
Pasture/crops (immediate)	2016	20.31	+ 2.30	1.09	− 0.16
	2019	22.61		0.93	
Developed (upstream)	2016	7.03	+ 1.33	3.56	+ 0.35
	2019	8.36		3.91	
Forest (upstream)	2016	60.14	− 3.06	84.26	− 0.01
	2019	57.08		84.25	
Pasture/crops (upstream)	2016	31.55	+ 1.60	8.44	+ 0.29
	2019	33.15		8.73	
# septic systems (immediate)	2017	8	–-	58	–-
# septic systems (upstream)		2053	–-	6896	–-
# cows in CAFOs (upstream)	2019	0	–-	356	–-
# swine in CAFOs (upstream)	2019	2753	–-	14,610	–-
# poultry in CAFOs (upstream)	2019	100,023	–-	0	–-

Pine, likely due to a more rural landscape setting, had a greater number of septic systems (nonpoint source) at both catchment scales (Table 2). Differences in point sources, such as confined animal feeding operations (CAFOs), were also identified. In the upstream catchment at Pine, there were a greater number of heads of cattle and swine in CAFOs (356 and 14,610, respectively), while at WBM, there were over 100,000 heads of poultry in CAFOs and none at Pine (Table 2).

At WBM, a higher concentration of total pesticide application in the immediate catchment was observed; however, in the upstream catchment, it was not always higher than in Pine (which had higher levels in 2015, 2016, 2018, and 2019). WBM had greater phytoestrogen crop cover at both catchment scales throughout the study. Despite Pine having more forested land cover, there were more NPDES facilities and higher concentrations of total nutrients from biosolids at both catchment scales (Table 3).

**Table 3:** Total estimated pesticide application, % high phytoestrogen crop cover, number (#) of industrial and municipal National Pollutant Discharge Elimination System (NPDES) facilities, and total nutrients from applied biosolids (kg) in the immediate and upstream catchments of West Branch Mahantango Creek (WBM) and Pine Creek (Pine) from 2015 to 2019

Land use variable	Year	WBM	Pine
Total pesticide application (kg/year; immediate)	2015	56.16	0.03
	2016	52.47	0.18
	2017	56.68	0.02
	2018	47.54	0.76
	2019	64.47	0.32
Total pesticide application (kg/year; upstream)	2015	11,120.06	14,437.87
	2016	10,426.39	10,650.66
	2017	11,697.80	9438.52
	2018	12,048.26	12,288.05
	2019	11,218.27	11,846.07
% high phytoestrogen crop cover (immediate)	2015	2.53	0.00
	2016	1.61	0.01
	2017	2.90	0.00
	2018	4.88	0.00
	2019	2.82	0.01
% high phytoestrogen crop cover (upstream)	2015	4.49	0.08
	2016	4.46	0.11
	2017	4.89	0.11
	2018	5.01	0.11
	2019	5.62	0.09
# NPDES facilities (upstream)	2015	12	78
	2016	15	83
	2017	19	88
	2018	24	95
	2019	26	99
Total nutrients from biosolids (kg/year; immediate)	2015	2.38	2.47
	2016	2.37	2.46
	2017	2.36	2.47
	2018	2.37	2.48
	2019	2.37	2.48
Total nutrients from biosolids (kg/year; upstream)	2015	809.35	2633.93
	2016	805.75	2615.17
	2017	802.50	2599.50
	2018	803.30	2613.24
	2019	804.10	2626.61

### Water chemistry—site comparisons

**Fig. 2:**
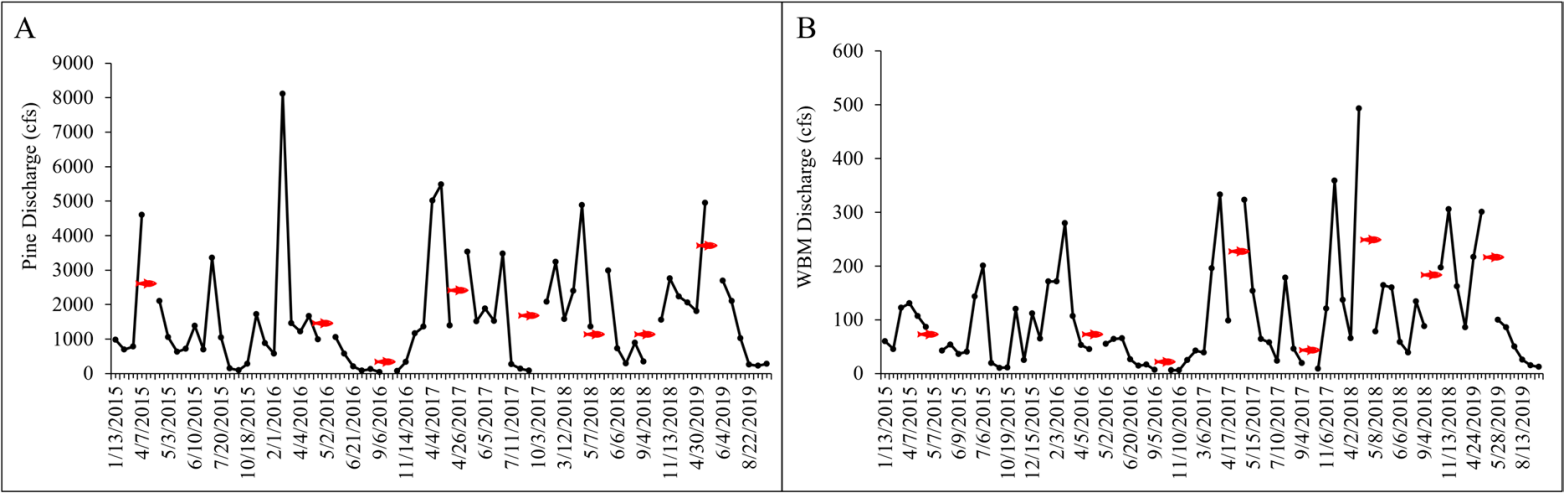
Flow (discharge) in 2015–2019 at the Pine Creek collection site (**A**) and the West Branch Mahantango Creek (WBM; **B**) site. Fish icon in red color indicates when fish were sampled

The following data are available in Walsh et al. (2024). For all years, the mean discharge at Pine in the spring (March–May) was 2314 ft^3^/s (cfs) and in the fall (September–November) was 684 cfs. At WBM, discharge rates were substantially lower, with a mean discharge rate of 142 cfs in the spring and 62 cfs in the fall. Discharge rates generally peaked in spring and decreased over the summer–winter months (Fig. 2A, B), although in 2017 and 2018, there were some high flows in the fall.

In many instances, peaks in contaminant concentrations occurred during or just after peaks in discharge rates (see figures listed below).

Table 4 lists the types and occurrence of contaminants detected at Pine and WBM. Of the 20 hormones and sterols (indicators of animal and human waste) analyzed, five were detected at least once at each site.

**Table 4:** The occurrence of contaminants detected at Pine Creek and West Branch Mahantango Creek (WBM) in 2015–2019, including hormones, phytoestrogens, current-use pesticides, mycotoxins, and wastewater/pharmaceutical contaminants

Contaminant type	Site	# of sampling occasions	Contaminant	# of detections	% detected
Hormones	Pine	58	17-alpha-estradiol	1	1.7
			17-alpha-ethynylestradiol	1	1.7
			3-beta-coprostanol	1	1.7
			Trans-diethylstilbestrol	1	1.7
			Cholesterol	26	44.8
	WBM	62	Cis-androsterone	1	1.6
			Estrone	1	1.6
			17-alpha-estradiol	3	4.8
			3-beta-coprostanol	14	22.6
			Cholesterol	53	85.5
Phytoestrogens	Pine	40	Biochanin	1	2.5
			Daidzein	4	10.0
			Equol	4	10.0
			Formononetin	7	17.5
			Genistein	18	45.0
	WBM	61	Coumesterol	1	1.6
			Biochanin	14	23.0
			Genistein	38	62.3
			Daidzein	39	63.9
			Equol	54	88.5
			Formononetin	57	93.4
Current-use pesticides	Pine	65	Simazine	2	3.1
			Metolachlor	16	24.6
			Atrazine	31	47.7
	WBM	68	Simazine	22	32.4
			Metolachlor	59	86.8
			Atrazine	63	92.6
Mycotoxins	Pine	No detections			
	WBM	47	Deoxynivalenol	3	6.4
			Nivalenol	4	8.5
			Beauvericin	5	10.6
			Zearalenone	9	19.1
Wastewater/pharmaceuticals	Pine	16	Caffeine	1	6.3
			Desmethylditiazem	1	6.3
			Fexofenadine	1	6.3
			Carbamazepine	2	12.5
			Desvenlafaxine	2	12.5
			Lidocaine	2	12.5
			Methocarbamol	2	12.5
			Tramadol	2	12.5
			Nicotine	7	43.8
			Metformin	12	75.0
	WBM	16	Hexamethylenetetramine	1	6.3
			Gabapentin	1	6.3
			Piperonyl butoxide	1	6.3
			Carbamazepine	1	6.3
			Sulfamethoxazole	1	6.3
			Trimethoprim	1	6.3
			Prednisolone	1	6.3
			Methocarbamol	1	6.3
			Sulfadimethoxine	1	6.3
			Methotrexate	1	6.3
			Desmethyldiltiazem	1	6.3
			Acetaminophen	2	12.5
			Metoprolol	2	12.5
			Venlafaxine	2	12.5
			Cotinine	3	18.8
			Tramadol	4	25.0
			Fexofenadine	6	37.5
			Lidocaine	7	43.8
			Nicotine	7	43.8
			Desvenlafaxine	7	43.8
			Caffeine	8	50.0
			Metformin	15	93.8

**Fig. 3:**
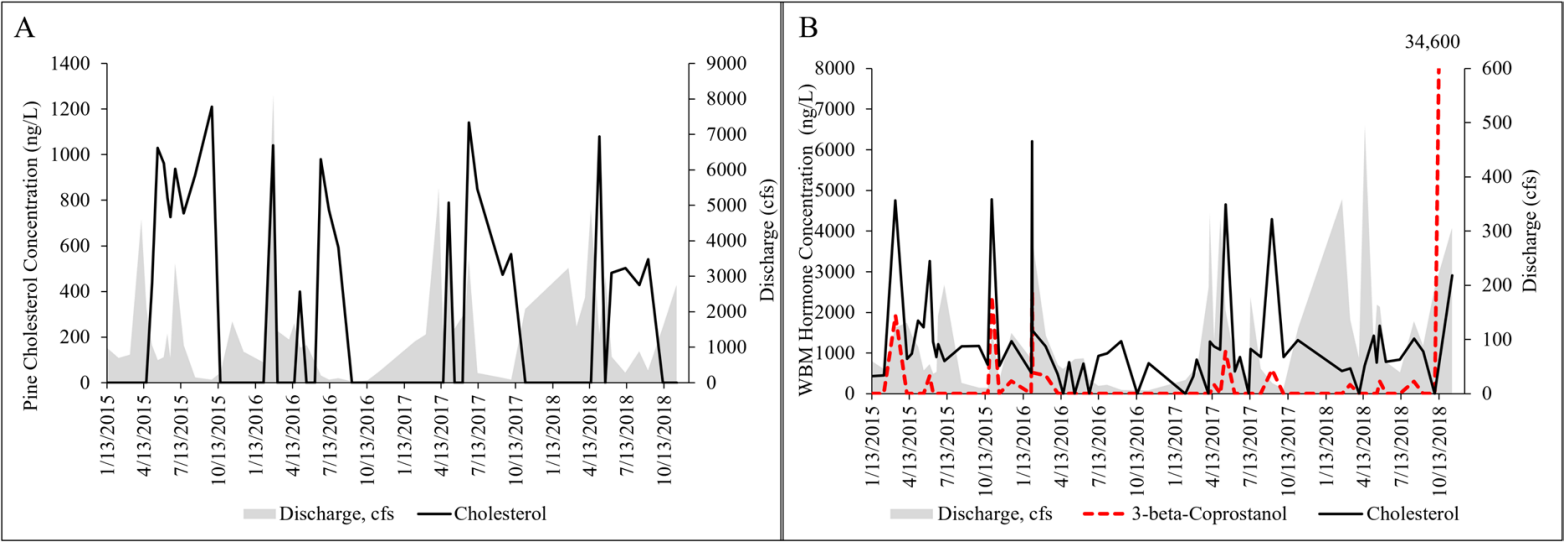
Concentrations of the most detected sterol hormones, including cholesterol at Pine Creek (**A**) and cholesterol and 3-beta-coprostanol at West Branch Mahantango Creek (WBM; **B**) in 2015–2019

At Pine, five hormones were detected, including 17-alpha-estradiol, 17-alpha-ethynylestradiol, 3-beta-coprostanol, trans-diethylstilbestrol, and cholesterol. At WBM, five hormones were also detected including cis-androsterone, estrone, 17-alpha-estradiol, 3-beta-coprostanol (with a high peak of 34,600 ng/L on 11/13/2018), and cholesterol. Cholesterol concentrations at Pine rarely exceeded 1000 ng/L, while levels at WBM were often above that and reached 6000 ng/L (Fig. 3).

**Fig. 4:**
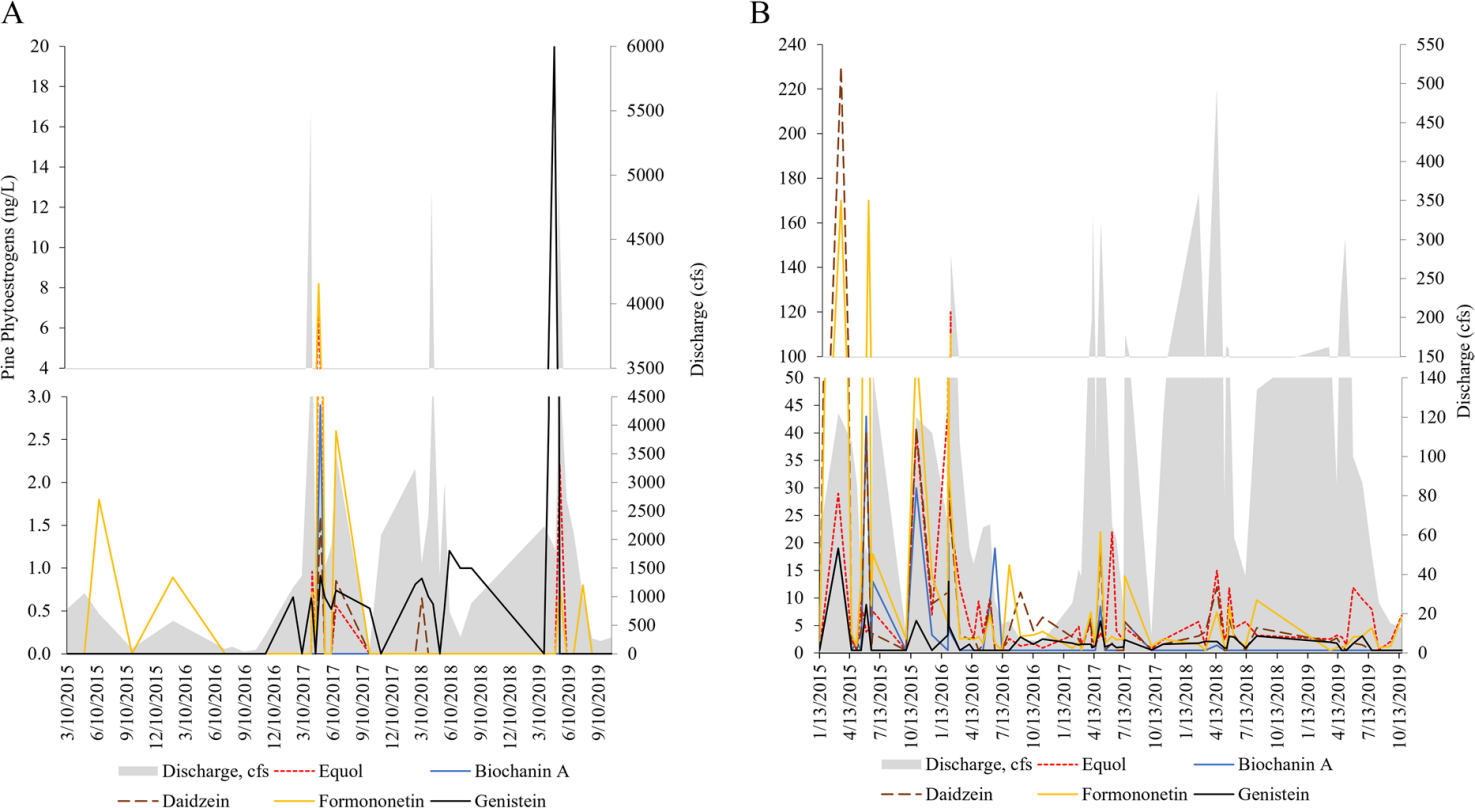
Concentrations of the five most detected phytoestrogens at **A** Pine Creek and **B** West Branch Mahantango Creek (WBM) in 2015–2019

Phytoestrogens, including the isoflavones biochanin, coumesterol, daidzein, equol, formononetin, and genistein occurrence and concentrations, were higher at WBM than at Pine (Fig. 4A, B) and fluctuated throughout the year. Of these, five were detected at Pine including biochanin, daidzein, equol, formononetin, and genistein.

At WBM, six phytoestrogens were detected including coumesterol, biochanin, genistein, daidzein, equol, and formononetin.

**Fig. 5:**
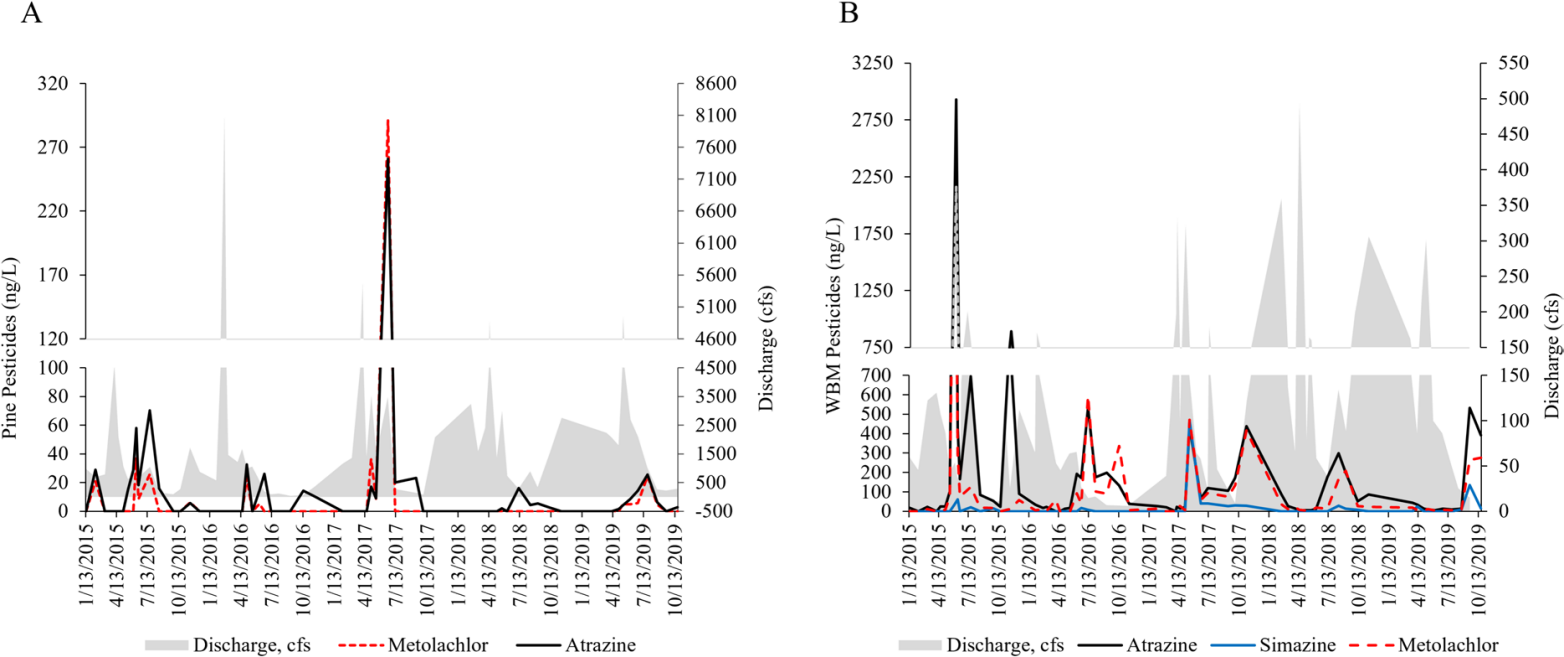
Concentrations of the most detected pesticides, including metolachlor and atrazine at Pine Creek (**A**) and atrazine, simazine, and metolachlor at West Branch Mahantango Creek (WBM; **B**) in 2015–2019

Of the 105 current-use pesticides analyzed, three were commonly detected, including atrazine, metolachlor, and simazine. Like phytoestrogens, the concentrations (Fig. 5A, B) and occurrence of these three pesticides were lower at Pine than at WBM.

Peaks in late spring and summer were often observed. The highest concentrations of atrazine and metolachlor were observed on 6/20/2017 and were 261 ng/L and 291 ng/L, respectively. For most of the sampling events at Pine, atrazine concentrations were below 50 ng/L and metolachlor below 30 ng/L. At WBM, the high peaks of atrazine and metolachlor occurred on 6/1/2015 and were 2930 ng/L and 2160 ng/L, respectively. While there were peaks in spring/summer at WBM, peaks in the fall (primarily atrazine and metolachlor) were also observed.

**Fig. 6:**
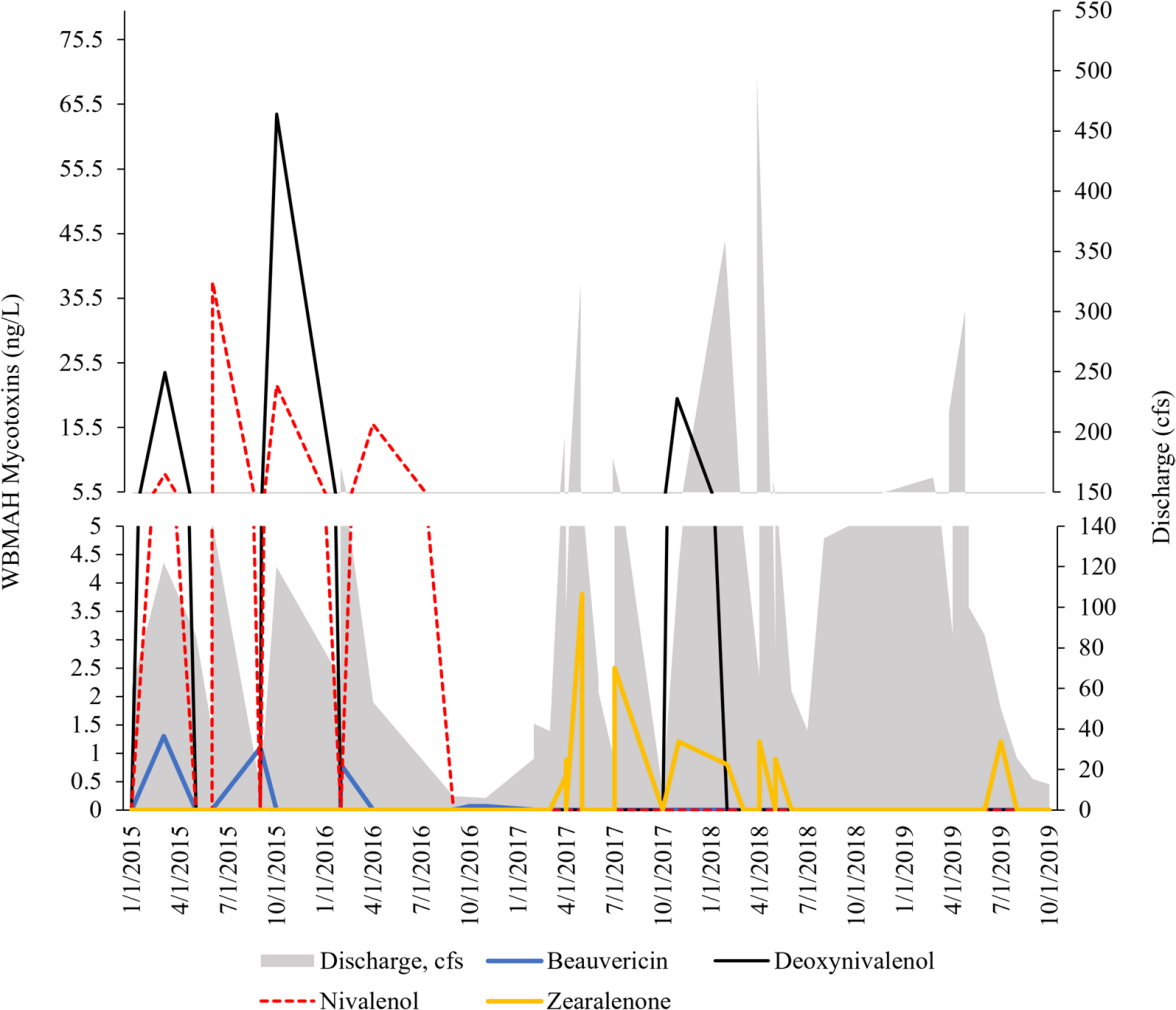
Concentrations of the mycotoxins detected at West Branch Mahantango Creek (WBM) in 2015–2019. No mycotoxins were detected at Pine Creek

Of the eight mycotoxins sampled during this study, none were detected at Pine. At WMB, deoxynivalenol, nivalenol, beauvericin, and zearalenone were detected. The mycotoxin with the highest peak was deoxynivalenol (64 ng/L on 10/29/2015) followed by nivalenol (38 ng/L on 6/21/2015; Fig. 6).

Even though zearalenone had the greatest number of detects, it was not detected until spring 2017, and beauvericin was not detected after 2016.

**Fig. 7:**
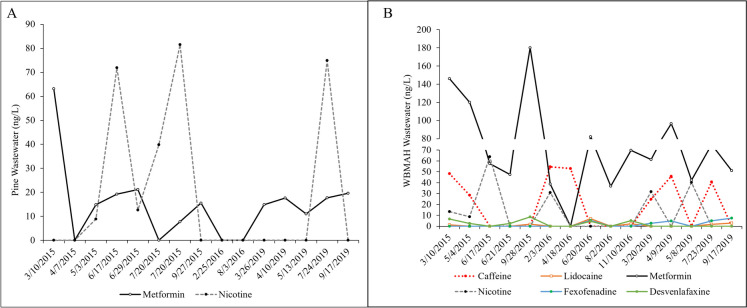
Concentrations of the most detected pharmaceuticals (wastewater indicator) at **A** Pine Creek and **B** West Branch Mahantango Creek (WBM) in 2015–2019

Wastewater was only sampled once in 2014 and occasionally in 2015, 2016, and 2019 for a total of 16 occasions at each site (Fig. 7).

At Pine, 10 wastewater contaminants were detected, including caffeine, desmethyldiltiazem, fexofenadine, carbamazepine, desvenlafaxine, lidocaine, methocarbamol, tramadol, nicotine, and metformin. At WBM, more than double the number of wastewater contaminants were detected, including hexamethylenetetramine, gabapentin, piperonyl butoxide, carbamazepine, sulfamethoxazole, trimethoprim, prednisolone, methocarbamol, sulfadimethoxine, methotrexate, desmethyldiltiazem, acetaminophen, metoprolol, venlafaxine, cotinine, tramadol, fexofenadine, lidocaine, nicotine, desvenlafaxine, caffeine, and metformin. At both sites, metformin was the mostly commonly detected pharmaceutical and peaked at WBM at 180 ng/L on 9/28/2015 and at 63 ng/L on 3/10/2015 at Pine.

At Pine, the only contaminants that were detected > 4 times in a year for > 4 years were atrazine, and it was negatively associated (*p* < 0.001, rho =  − 1.00) with total pesticide application (immediate). At WBM, contaminants including formononetin, equol, atrazine, and metolachlor were detected > 4 times in a year for > 4 years. At WBM, formononetin was negatively associated with # NPDES facilities (upstream; (*p* < 0.001, rho =  − 1.00)) and positively associated (*p* < 0.001, rho = 1.00) with total pesticide application (upstream).

### Smallmouth bass biological variables—site comparisons

The following data are available in Walsh et al. (2024). Means ± standard error of the mean of the biological variables, including age, total length, weight, Ktl, HSI, and HAI, are provided in Supplementary Table 1.

Supplementary Table 1. Sample size, morphometric, and age data for smallmouth bass *Micropterus dolomieu* collected in 2015–2019 in the spring and fall from Pine Creek and West Branch Mahantango Creek (WBM). Data is presented as the mean ± standard error of the mean.

For most sampling occasions, 20 bass were collected for a total of 150 and 152 SMB at Pine and WBM, respectively (Supplementary Table 1). Although SMB were not older, they were significantly larger at WBM (mean weight = 584.58 gm, total length = 399.78 mm) than at Pine (mean weight = 387.01 gm, total length = 290.32 mm; *p* < 0.001). With all years and seasons combined, SMB from WBM (mean Ktl = 1.32) had a higher Ktl than Pine (mean Ktl = 1.27; *p* < 0.001), and HSI was only greater at WBM in the fall (mean HSI WBM = 1.09, Pine = 1.00; *p* = 0.008). HAI was not different between the two sites.

The mean results of parasite and macrophage aggregate densities in SMB are listed in Supplementary Table 2.

Supplementary Table 2. Liver and spleen parasite and macrophage aggregate (MA) densities from smallmouth bass *Micropterus dolomieu* collected in 2015–2019 in the spring and fall from Pine Creek and West Branch Mahantango Creek (WBM). Data is presented as the mean ± standard error of the mean.

With all years and seasons combined, parasite densities were significantly greater at Pine (mean in liver = 22.44 parasites/mm^2^, spleen = 12.26 parasites/mm^2^) than WBM (mean in liver = 7.87 parasites/mm^2^, spleen = 5.15 parasites/mm^2^; *p* < 0.001), and there were no significant differences in MA densities between the two sites.

### Gene transcript abundance and contaminant correlations

There were 10 gene transcripts (*arg*, *bcl2*, *c3*, *cyp1a*, *dio1*, *glk*, *hsp70*, *hsp90b*, *mt*, and *tf*) that were significantly different between seasons and 10 (*cat*, *gp*, *gr*, *gst*, *hprt1*, *ppar*, *sod*, *tgfβ*, *wap65*, and *dio2*) that were not at both sites*.* Additionally, there were gene transcripts that were different at each site but not different between seasons, including *tbp*, *crpl*, *pepck*, *hep2*, *gsr*, *thrb*, and *apa1* at WBM and *eh1*, *cyp3a*, *igf1*, and *pcna* at Pine. These were also included in the correlation analyses for each site, and transcripts that were different between the seasons were not included. Table 5 lists the results of the associations between gene transcripts and the most detected contaminants and/or land use variables at WBM and Pine.

**Table 5:** Annual land use variables and contaminants (2015–2019) significantly associated with smallmouth bass *Micropterus dolomieu* hepatic gene transcript abundance at West Branch Mahantango Creek (WBM) and Pine Creek (Pine). A Spearman’s rank correlation analysis with a Holm’s correction was used, and *p* < 0.05 was considered statistically significant

Site	Contaminant/land use	Gene transcript	Related function	*p* (Spearman’s rho)
WBM (males)	Formononetin	*tgfβ*	Immune/inflammation	< 0.001 (− 1.00)
		*cat*	Oxidative stress	< 0.001 (− 1.00)
	Pesticide application (upstream; kg/yr)	*ppar*	Lipid metabolism	< 0.001 (1.00)
	Phytoestrogen crop cover (immediate; %)	*ppar*	Lipid metabolism	< 0.001 (1.00)
	Phytoestrogen crop cover (upstream; %)	*wap65*	Stress	< 0.001 (1.00)
	Total nutrients from biosolids (upstream; kg/yr)	*sod*	Oxidative stress	< 0.001 (− 1.00)
		*gsr*	Oxidative stress	< 0.001 (− 1.00)
	# NPDES facilities (upstream)	*tgfβ*	Immune/inflammation	< 0.001 (1.00)
		*cat*	Oxidative stress	< 0.001 (1.00)
WBM (females)	Equol	*hep2*	Immune/inflammation	< 0.001 (− 1.00)
Pine (males)	# NPDES facilities (upstream)	*cat*	Oxidative stress	< 0.001 (1.00)
	Pesticide application (immediate; kg/yr)	*dio2*	Thyroid	< 0.001 (− 1.00)
Pine (females)	Pesticide application (upstream; kg/yr)	*dio2*	Thyroid	< 0.001 (1.00)
	Total nutrients from biosolids (upstream; kg/yr)	*hprt1*	Cell proliferation	< 0.001 (1.00)
		*eh1*	Contaminant metabolism	< 0.001 (1.00)
	# NPDES facilities (upstream)	*tgfβ*	Immune/inflammation	< 0.001 (1.00)

Overall, there were more associations between contaminants/land use variables and gene transcripts at WBM (10 associations) than at Pine (6 associations), and at both sites, sex differences were observed. At WBM, only formononetin, equol, atrazine, and metolachlor had enough detections within a week prior to fish sampling that could be used in statistical analyses. In females, there was only one association between an immune/inflammation-related transcript (*hep2*) and equol. In males, *tgfβ* (immune/inflammation) and *cat* (oxidative stress) were negatively associated with formononetin but positively associated with # NPDES facilities (upstream). Pesticide application (upstream) and phytoestrogen crop cover (immediate) were both positively associated with the lipid metabolism transcript, *ppar*, and two other oxidative stress transcripts, *sod* and *gsr*, were negatively associated with total nutrients from biosolids (upstream). Phytoestrogen crop cover (upstream) was positively associated with the stress-related transcript, *wap65*.

At Pine, atrazine was the only contaminant with enough observations that could be used in the correlation analyses; however, it was not associated with any transcripts. In males, *cat* (oxidative stress) was positively associated with # NPDES facilities (upstream), and *dio2* (thyroid) was negatively associated with pesticide application (immediate). Unlike the sex differences observed at WBM, females at Pine had more associations than males. Pesticide application (upstream) was positively associated with *dio2* (thyroid), total nutrients from biosolids were positively associated with *hprt1* (cell proliferation) and *eh1* (contaminant metabolism), and # NPDES facilities (upstream) were positively associated with *tgfβ* (immune/inflammation). There were no transcripts in either sex associated with phytoestrogen crop cover.

### Biological variable and contaminant correlations

At both sites, MA density was the only variable associated with contaminants/land use. At WBM, phytoestrogen crop cover (immediate and upstream) was negatively associated (*p* < 0.001, rho =  − 1.00) with spleen and liver MA density, respectively, and total pesticide application (upstream) was negatively associated with spleen MA density (*p* < 0.001, rho =  − 1.00). At Pine, only # NPDES facilities (upstream) were negatively associated with liver MA density (*p* < 0.001, rho =  − 1.00). There were no associations with Ktl, HSI, HAI, or parasite density.

### Principal component analysis and multiple linear regression

**Fig. 8:**
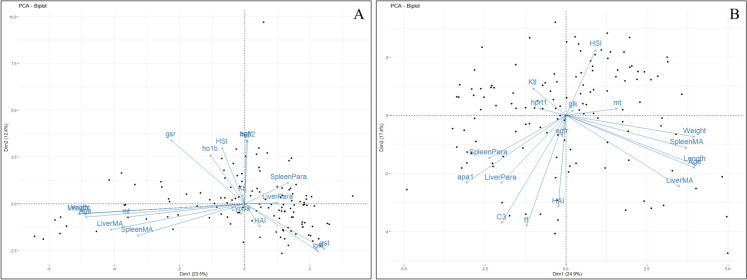
PCA biplots of biological variables of smallmouth bass (*Micropterus dolomieu*) including size (total length and weight), age, condition factor (Ktl), hepatosomatic index (HSI), health assessment index (HAI), liver and spleen macrophage aggregate (MA) and parasite (Para) density (#/mm.^2^), and hepatic gene transcripts with factor loadings ≥ 0.3 from Pine Creek (**A**) and West Branch Mahantango Creek (WBM; **B**)

Figure 8 displays PCA biplots for each site that include age, weight, length, parasite and MA density, Ktl, HAI, HSI, and gene transcripts with factor loadings ≥ 0.3.

At Pine, PC1 explained 23.5% of the variation among samples, and the variables with the most influence were size (length and weight), age, MA density, and *mt*. At WBM, PC1 explained 24.9% of the variance and was most influenced by HAI, *tf*, *c3*, *apa1*, size (length and weight), age, and MA density. The mlr models were useful for identifying significant relationships between gene transcripts with factor loadings ≥ 0.3, biological variables, season, year, and sex (Supplementary Tables 3, 4, 5, 6, 7, and 8). At WBM, HSI had a positive relationship with season (spring > fall) and *hprt1* (cell proliferation) and had a negative relationship with year and sex (F > M). HAI was positively related to age, *apa1* (lipid metabolism), and *tf* (immune/inflammation) and negatively related to *c3* (immune/inflammation; Supplementary Table 3).

Supplementary Table 3. Results of the best fit multiple linear regression (mlr) models used to identify significant relationships between hepatic gene transcripts, season, year, age, and smallmouth bass *Micropterus dolomieu* hepatosomatic index (HSI) and the health assessment index (HAI) at West Branch Mahantango Creek (WBM) in the spring and fall 2015–2019. *P* < 0.05 was considered statistically significant.

At Pine, *gsr* (oxidative stress), season, and year were positively related to HSI, and sex was negatively related (females > males). HAI was positively related to year and age (Supplementary Table 4).

Supplementary Table 4. Results of the best fit multiple linear regression (mlr) models used to identify significant relationships between hepatic gene transcripts, season, year, age, and smallmouth bass *Micropterus dolomieu* hepatosomatic index (HSI) and the health assessment index (HAI) Pine Creek (Pine) spring and fall 2015–2019. *P* < 0.05 was considered statistically significant.

At WBM, liver parasite density was negatively related to season (spring < fall), and spleen parasite density was negatively related to age (Supplementary Table 5).

Supplementary Table 5. Results of the best fit multiple linear regression (mlr) models used to identify significant relationships between hepatic gene transcripts, season, year, age, and smallmouth bass *Micropterus dolomieu* liver and spleen parasite density at West Branch Mahantango Creek (WBM) in the spring and fall 2015–2019. *P* < 0.05 was considered statistically significant.

Liver and spleen MA density were both positively related to age (Supplementary Table 6); however, only liver MA density was negatively related to season (spring < fall).

Supplementary Table 6. Results of the best fit multiple linear regression (mlr) models used to identify significant relationships between hepatic gene transcripts, season, year, age, and smallmouth bass *Micropterus dolomieu* liver and spleen macrophage aggregate (MA) density at West Branch Mahantango Creek (WBM) in the spring and fall 2015–2019. *P* < 0.05 was considered statistically significant.

While there was not a significant relationship with year, it is interesting to note that both liver and spleen MA densities generally decreased over time in the spring and increased over time in the fall (Supplementary Table 2). There were no hepatic transcripts significantly related to a parasite or MA density.

At Pine, there were no significant relationships with liver or spleen parasite densities (Supplementary Table 7).

Supplementary Table 7. Results of the best fit multiple linear regression (mlr) models used to identify significant relationships between hepatic gene transcripts, season, year, age, and smallmouth bass *Micropterus dolomieu* liver and spleen parasite density at Pine Creek (Pine) in the spring and fall 2015–2019. *P* < 0.05 was considered statistically significant.

Although not significant, parasite densities also showed a decrease with year and age. On the contrary, liver and spleen MA densities were positively related to age, and liver MA density significantly decreased over time (Supplementary Table 8).

Supplementary Table 8. Results of the best fit multiple linear regression (mlr) models used to identify significant relationships between hepatic gene transcripts, season, year, age, and smallmouth bass *Micropterus dolomieu* liver and spleen macrophage aggregate (MA) density at Pine Creek (Pine) in the spring and fall 2015–2019. *P* < 0.05 was considered statistically significant.

## Discussion

Integrated fish health assessments, which incorporate land use, water chemistry, and biological variables (from the molecular to the organismal levels), have the capability to provide information on the effects of contaminant exposure on individuals and potential population-level effects. Pollutants are known to alter gene expression in fish, making transcriptional changes a sensitive endpoint for examining exposure effects (Bozinovic & Oleksiak, 2011). The aim of the current study was to use a multi-level approach to investigate effects and differences on certain molecular, cellular, and tissue endpoints in smallmouth bass at a site with agricultural/developed land use compared to a site with predominantly forested land use. At the agriculturally impacted site, WBM; contaminants such as hormones, phytoestrogens, pesticides, mycotoxins; and wastewater were detected more often and at higher concentrations than at the forested site (Pine). Land use variables at WBM also included greater total pesticide application and phytoestrogen crop cover. At WBM, more contaminant and land use associations with hepatic gene transcripts were observed in SMB than at Pine. In regions with chronic pollution, an increase in the alteration of expressed genes has been observed (Oleksiak, 2008) which could explain why there were more changes in gene transcript abundance at WBM than at Pine.

There are many factors known to influence water quality, including stream size and land cover (Lintern et al., 2018). The upstream and immediate catchment size of the Pine Creek site was substantially larger, and discharge rates were higher (up to 16 × greater in the spring than WBM) which could dilute contaminant loads. However, WBM had more than 100,000 heads of poultry in CAFOs in the upstream catchment and around 20% more pasture/crop land cover in the immediate catchment than Pine. In a groundwater study conducted at WBM and Pine, higher concentrations of pesticides and phytoestrogens were detected at WBM (Thompson et al., 2021), indicating groundwater may be a secondary contaminant source besides surface runoff. Average total pesticide application rates were substantially higher in the immediate catchment of WBM (> 200 ×); however, in the upstream catchment, they were similar. Watershed scale has been shown to have a major influence on the effects of landscape variables on SMB, especially at the immediate scale (Blazer et al., 2021). Amendments, such as fertilizers and organic matter, can play a role in the physiochemical nature of contaminants by impacting processes such as adsorption–desorption, bioavailability, and mobility (Carpio et al., 2021). Pesticide degradation, including abiotic (chemical and photochemical reactions) and biotic (microorganisms or plants), also plays a role in pesticide availability and transport within catchments (Wang et al., 2019); thus, analysis at a smaller scale is likely to capture the presence of parent compounds before they breakdown. Only at Pine was pesticide application in the immediate catchment associated with an individual pesticide (atrazine), which may be because individual pesticides were detected at discrete time points, and total pesticide application is an annual total that accounts for all pesticide compounds applied in a given state for that year. Temporal fluctuations in contaminant concentrations were also observed at each site and peaks often coincided with, or followed, high-flow events or times of low flow. Similar trends were observed at another agriculturally impacted site sampled during the same years in the Potomac River, Maryland (also located in the Chesapeake Bay watershed; Walsh et al., 2022). Thus, it is critical to understand the impact of flow, landscape drivers, and timing of pesticide, fertilizer, and cover crop applications on temporal exposure patterns.

In fish and other organisms, phytoestrogens are known endocrine disruptors that can have reproductive (Cleveland & Manor, 2015; Latonnelle et al., 2002; Stevenson et al., 2011), growth (Cleveland & Manor, 2015), and other health-related effects (Coltfelter and Rodriguez, 2006; Schiller et al., 2013; Yang et al., 2022). At WBM, phytoestrogen crop cover in the immediate and upstream catchment was between 0.14 and 2.04% higher, respectively, than at Pine. In male SMB at WBM, phytoestrogen crop cover was positively associated with *wap65* (stress) and *ppar* (lipid metabolism). In fish, *wap65* has been shown to be upregulated in response to contaminant exposure (Blazer et al., 2023; Pierre et al., 2011) and is believed to have a function similar to *hsp70* in responding to environmental and thermal stressors (Pierre et al., 2011). In addition to phytoestrogen crop cover, *ppar* was also positively associated with pesticide application (upstream) and has been shown to affect carbohydrate (glucose) metabolism and energy homeostasis (Ning et al., 2016) and reproduction (Sritharan, 2017). Male SMB in the Chesapeake Bay drainage have historically demonstrated a high rate of intersex (Blazer et al., 2014) in response to estrogenic contaminant exposure; thus, this information provides a deeper understanding of potential mechanisms involved in this response that warrant further study.

In males at WBM, there were a greater number of associations between gene transcripts and contaminants/land use variables, while at Pine, females had more. Additionally, there were no associations with phytoestrogen crop cover at Pine. Factors such as excretion methods (ex. eggs in females and milt in males; Burger, 2007) and hormone modulation (Liu et al., 2015) may help explain how and why sex differences occur. Regardless, it is not clear what health or reproductive effects these differences may have. While some studies have found beneficial effects of phytoestrogen isoflavones on growth, immunity, and disease resistance (Pastore et al., 2018; Zhou et al., 2015), others have identified altered behavioral patterns (Clotfelter & Rodriguez, 2006) and impaired sexual development (Kiparissis et al., 2003). At Pine, both males and females showed associations between the thyroid-related transcript, *dio2*, and pesticide application; however, in males, the association was negative, and in females, the association was positive. In fish, *dio2* is important in thyroid regulation, particularly during reproduction. In zebrafish, a disruption in *dio2* production was shown to have deleterious reproductive effects in both sexes (Houbrechts et al., 2019). Understanding how contaminant exposure differentially affects the sexes and impacts hormone regulation will be important to understand reproductive effects during spawning and recrudescence.

As part of the immune response, MA are involved in the detoxification of foreign substances and iron recycling (Steinel & Bolnick, 2017; Wolke, 1992) and are considered useful indicators of exposure to environmental contamination (Badamasi et al., 2019; Blazer et al., 1987; Fournie et al., 2001). At both sites, negative associations were observed between MA densities and certain types of land use including phytoestrogen crop cover and total pesticide application at WBM and # NPDES facilities at Pine. Neither season nor hepatic gene transcripts were associated with MA density at Pine; however, at WBM, MA density was negatively related to season (with a decrease in spring). Studies have shown that fish exhibit MA differences due to age, sex, weight, season, and site (Blazer et al., 1987), exposure to pesticides and organic pollutants (Matsche et al., 2021), and parasitic infections (Matsche et al., 2019). At both sites in this study, age was positively associated with MA density. Most fish studies in the literature have found positive associations between contaminants and MA density (Blazer et al., 1987; Fournie et al., 2001; Matsche et al., 2021); however, some have found negative or no associations (Carreras-Colom et al., 2022; Haaparanta et al., 1996). In the spring, MA density in bass from WBM was lower and phytoestrogen (particularly formononetin, genistein, and equol) and pesticide concentrations often peaked. Laboratory studies would provide a better understanding of the role these exposures have on MA development and density in SMB and whether the negative associations observed in this study may be linked with an impaired immune response.

The HAI includes ratings of internal and external abnormalities based on the severity of related health effects. At some sites, HAI scores have been shown to be significantly associated with pollutants, including PCBs (Adams et al., 1993), mercury (Blazer et al., 2023), arsenic (Adams et al., 2010), and paper mill effluent (Kovacs et al., 2002); however, a study in an agricultural setting found no associations between HAI and improvements in agricultural practices (known as best management practices; Jensen, 2023). In this study, HAI was not significantly different between sites, but age was identified as a significant and positive predictor (similar to results observed in smallmouth bass; Jensen, 2023), and at Pine, year was also a positive predictor. Although the year was not related to HAI in bass from WBM, at both sites, HAI was highest in spring 2019. No land use variables were associated with HAI at either site; therefore, it is likely that the high HAI scores in 2019 were associated with a variable not measured in this study. In the liver, small white cysts and general and focal discoloration were the most common abnormalities recorded. At Pine, more occurrences of small white cysts (likely trematode metacercariae) were observed while at WBM, there was more general/focal discoloration. Only at WBM were hepatic gene transcripts, *apa1*, *tf*, and *c3*, related to HAI. Apolipoproteins are well known to play a role in lipid metabolism; however, they also function as part of the innate immune system (Sahoo et al. 2017) as well as *tf* (Stafford & Belosevic, 2003) and *c3* (Raposo de Magalhães et al., 2020). The differences in the liver abnormalities which contributed to HAI profiles at each site warrant further exploration. No hepatic gene transcripts were associated with parasites at Pine, which may be why none were associated with the HAI. However, a more in-depth assessment of gene transcripts associated with liver discoloration and the associated cellular changes at multiple sites may help explain the mechanisms involved.

Overall, a greater number of associations between contaminants and altered gene transcripts were identified at WBM which may be a consequence of higher and more frequent contaminant detections. On the contrary, mean parasite density was significantly higher at Pine, which was four and five times as high in the liver and spleen, respectively. Like fish, parasites are susceptible to environmental contaminants and come into direct contact with them in the water column or within a host (Khan & Thulin, 1991). The study of this interaction has been termed “environmental parasitology” (Sures, 2004), and digenean trematodes (observed in this study) are particularly useful indicators due to their multiple-host lifestyle rendering them susceptible to the repeated risk of contaminant exposure (Pietrock & Marcogliese, 2003). The lower parasite densities at WBM could have been associated with higher concentrations of contaminants, decreased host availability (snails and birds), and/or lower parasite survival. At Pine, parasite density was not related to any hepatic transcripts, season, year, age, or sex. It is also worthwhile to note that SMB at WBM were larger and had higher Ktl than SMB at Pine. The greater productivity in the waters of WBM, likely associated with more nutrient runoff, may explain why SMB were larger and more fit than at Pine and perhaps this increased their ability to prevent parasitic infections. Determining why smallmouth bass at Pine Creek had higher parasite densities would require an understanding of host availability/density and habitat suitability. It is difficult to say what role contaminants had on parasite density at these two sites and more work would be needed to effectively tease out these relationships.

Research has shown that the results obtained in gene expression studies in wild fishes are complex, and the selection of robust candidate biomarkers should be carefully selected (Tine, 2017). Studies by Sures (2004, 2006, 2007) recognize that parasitic infections can modify biomarkers (such as oxidative stress and contaminant metabolism endpoints) commonly used in teleost ecotox studies, potentially leading to biased results. Other factors such as age, sex, condition, reproductive status, and season can also cause incongruity (Marcogliese & Pietrock, 2011). One suggestion is to incorporate a multi-omics approach, including genomics, proteomics, and metabolomics, to fully understand how biological variables respond to environmental stressors (Kumar & Denslow, 2017). Although our smallmouth bass health assessments have become more comprehensive, the inclusion of an environmental omics (Ge et al., 2013) approach would be a beneficial next step forward. Lastly, while this was a multi-year study, the limited number of observations (5 years) may have resulted in false-positive relationships (Aggarwal & Ranganathan, 2016) due to a decrease in power and an overestimation of effect (Lazic, 2018). Thus, longer sampling periods over multiple years and seasons could increase the statistical power and lower variability.

## Conclusion

The integrated, fish health assessment approach presented in this study provides a better understanding of the effects agriculture-associated contaminants/land use have on smallmouth bass health endpoints from the molecular, cellular, and organismal levels. Not all types of contaminants were sampled consistently throughout the duration of the study, which limited the number of associations that could be analyzed with biological variables. More consistent surface water sampling and inclusion of liver tissue contaminant analysis, would help determine if contaminants detected in the water also bioaccumulate in the liver and are associated with transcriptional changes. Also, based on the changes observed in oxidative and contaminant metabolism-related gene transcripts, it would be worthwhile to include other contaminant analyses, including heavy metals. The results of this study identified the importance of timing for land management practices and how contaminants respond temporally to changes in flow. They also show that agricultural practices affect hepatic gene transcripts associated with immune function and disease resistance and may negatively affect the ability of smallmouth bass to resist opportunistic pathogens. Differences in gene transcript abundance associated with contaminants were observed between the sexes which is important information to consider for population management control. And while this work included many years of contaminant analyses, a better approach may also be to include a time-integrated sampling, such as the use of passive samplers, to get a deeper understanding of exposure patterns and biological responses. Additional work at these sites could include immune function, endocrine disruption, and additional gene transcript (gonad and anterior kidney) abundance analyses which will provide a broader scope of the health effects at Pine and WBM in addition to other sites within the Susquehanna River watershed.

## Supplementary Information

Below is the link to the electronic supplementary material.

Supplementary file1 (DOCX 21 KB)

Supplementary file2 (DOCX 19 KB)

Supplementary file3 (DOCX 15 KB)

Supplementary file4 (DOCX 15 KB)

Supplementary file5 (DOCX 15 KB)

Supplementary file6 (DOCX 15 KB)

Supplementary file7 (DOCX 14 KB)

Supplementary file8 (DOCX 15 KB)

## Data Availability

Data is provided through the U.S. Geological Survey ScienceBase at 10.5066/P13EHVRD.
