# Supplementary material for: Temporal analysis of water chemistry and smallmouth bass (Micropterus dolomieu) health at two sites with divergent land use in the Susquehanna River watershed, Pennsylvania, USA
Source: Environ Monit Assess. 2024 Sep 11;196(10):922. doi: 10.1007/s10661-024-13049-4 (PMC11390901; doi:10.1007/s10661-024-13049-4)
Supplement: Supplementary file 1 — Supplementary file1 (DOCX 21 KB) [file 10661_2024_13049_MOESM1_ESM.docx]

|  | Sampling Date | Sex | Age | Total Length (mm) | Weight (g) | Ktl | HSI | HAI |
| --- | --- | --- | --- | --- | --- | --- | --- | --- |
| Pine | 4/15/2015 | M (17) | 7.2 ± 0.93 | 393.1 ± 15.79 | 813.9 ± 91.94 | 1.23 ± 0.02 | 0.96 ± 0.04 | 42.4 ± 8.73 |
|  |  | F (2) | 6.5 ± 0.50 | 385.0 ± 10.00 | 708.5 ± 44.50 | 1.17 ± 0.01 | 1.74 ± 0.07 | 50.0 ± 20.00 |
|  | 5/2/2016 | M (13) | 6.1 ± 0.85 | 363.2 ± 17.20 | 684.2 ± 89.83 | 1.31 ± 0.02 | 1.04 ± 0.09 | 58.5 ± 9.66 |
|  |  | F (7) | 4.4 ± 0.48 | 343.3 ± 18.25 | 564.9 ± 86.68 | 1.27 ± 0.05 | 1.74 ± 0.20 | 54.3 ± 11.10 |
|  | 10/11/2016 | M (7) | 3.6 ± 0.20 | 222.0 ± 4.73 | 135.7 ± 7.93 | 1.23 ± 0.03 | 0.77 ± 0.05 | 74.3 ± 7.19 |
|  |  | F (9) | 3.4 ± 0.18 | 220.8 ± 4.41 | 135.1 ± 7.73 | 1.24 ± 0.03 | 1.03 ± 0.11 | 60.0 ± 6.87 |
|  | 4/26/2017 | M (8) | 5.9 ± 0.95 | 367.8 ± 27.58 | 768.0 ± 182.25 | 1.32 ± 0.05 | 1.13 ± 0.08 | 61.3 ± 13.15 |
|  |  | F (12) | 5.2 ± 0.55 | 330.4 ± 20.62 | 546.5 ± 112.44 | 1.21 ± 0.02 | 1.74 ± 0.13 | 51.7 ± 6.38 |
|  | 10/3/2017 | M (9) | 2.3 ± 0.17 | 226.9 ± 4.15 | 144.8 ± 8.33 | 1.22 ± 0.02 | 0.70 ± 0.04 | 45.6 ± 5.03 |
|  |  | F (11) | 2.5 ± 0.16 | 220.0 ± 6.53 | 138.7 ± 13.99 | 1.25 ± 0.03 | 0.79 ± 0.05 | 70.0 ± 9.72 |
|  | 5/14/2018 | M (13) | 4.2 ± 0.28 | 306.5 ± 11.66 | 401.8 ± 41.74 | 1.32 ± 0.03 | 1.22 ± 0.09 | 62.3 ± 8.78 |
|  |  | F (6) | 3.8 ± 0.40 | 237.3 ± 9.63 | 211.0 ± 22.91 | 1.47 ± 0.20 | 2.00 ± 0.13 | 48.3 ± 7.03 |
|  | 10/10/2018 | M (4) | 3.5 ± 0.29 | 251.5 ± 21.23 | 235.3 ± 52.99 | 1.36 ± 0.06 | 1.21 ± 0.17 | 65.0 ± 14.43 |
|  |  | F (14) | 4.4 ± 0.31 | 265.8 ± 9.19 | 250.4 ± 26.76 | 1.24 ± 0.02 | 1.38 ± 0.15 | 66.4 ± 10.77 |
|  | 5/29/2019 | M (16) | 3.3 ± 0.24 | 305.7 ± 8.97 | 393.4 ± 34.05 | 1.33 ± 0.03 | 1.16 ± 0.04 | 98.1 ± 6.07 |
|  |  | F (2) | 4.5 ± 1.50 | 357.5 ± 62.50 | 683.5 ± 333.50 | 1.23 ± 0.02 | 2.01 ± 0.06 | 115.0 ± 55.00 |
| WBM | 5/7/2015 | M (7) | 3.9 ± 0.26 | 369.6 ± 19.14 | 778.6 ± 115.95 | 1.46 ± 0.08 | 0.85 ± 0.05 | 64.3 ± 12.70 |
|  |  | F (10) | 3.9 ± 0.28 | 375.5 ± 13.94 | 840.9 ± 80.05 | 1.41 ± 0.03 | 1.82 ± 0.08 | 52.0 ± 11.43 |
|  | 4/25/2016 | M (8) | 5.0 ± 0.38 | 398.8 ± 14.78 | 959.5 ± 78.25 | 1.50 ± 0.07 | 0.70 ± 0.07 | 82.5 ± 16.56 |
|  |  | F (12) | 4.6 ± 0.19 | 407.1 ± 8.04 | 1,036.2 ± 63.51 | 1.39 ± 0.03 | 1.42 ± 0.11 | 55.8 ± 9.65 |
|  | 10/11/2016 | M (11) | 2.4 ± 0.16 | 195.8 ± 6.86 | 100.3 ± 10.67 | 1.29 ± 0.04 | 1.39 ± 0.09 | 68.2 ± 8.72 |
|  |  | F (6) | 2.3 ± 0.21 | 182.5 ± 3.28 | 79.2 ± 4.00 | 1.30 ± 0.03 | 1.15 ± 0.11 | 50.0 ± 7.30 |
|  | 4/20/2017 | M (10) | 3.9 ± 0.41 | 353.6 ± 14.10 | 662.3 ± 79.29 | 1.43 ± 0.06 | 0.98 ± 0.04 | 48.0 ± 10.20 |
|  |  | F (10) | 4.7 ± 0.47 | 388.8 ± 15.49 | 862.2 ± 96.49 | 1.31 ± 0.03 | 1.69 ± 0.10 | 43.0 ± 11.26 |
|  | 10/2/2017 | M (12) | 3.9 ± 0.69 | 410.4 ± 66.32 | 489.4 ± 88.20 | 1.16 ± 0.15 | 1.06 ± 0.09 | 70.0 ± 9.77 |
|  |  | F (8) | 2.3 ± 0.16 | 269.0 ± 15.00 | 294.9 ± 47.72 | 1.40 ± 0.05 | 1.05 ±0.05 | 68.8 ± 8.54 |
|  | 5/7/2018 | M (9) | 4.3 ± 0.29 | 350.9 ± 6.61 | 535.0 ± 40.06 | 1.23 ± 0.07 | 0.99 ± 0.15 | 40.0 ± 8.98 |
|  |  | F (10) | 3.9 ± 0.10 | 352.8 ± 4.49 | 605.2 ± 26.76 | 1.26 ± 0.03 | 1.47 ± 0.05 | 35.0 ± 8.60 |
|  | 10/2/2018 | M (9) | 6.0 ± 0.82 | 373.0 ± 16.34 | 748.9 ± 102.07 | 1.36 ± 0.02 | 0.87 ± 0.07 | 78.9 ± 15.67 |
|  |  | F (11) | 5.9 ± 0.67 | 378.4 ± 17.89 | 811.5 ± 107.49 | 1.37 ± 0.04 | 1.02 ± 0.08 | 70.9 ± 8.47 |
|  | 5/28/2019 | M (16) | 4.1 ± 0.24 | 358.8 ± 7.64 | 642.8 ± 34.09 | 1.37 ± 0.02 | 0.92 ± 0.04 | 111.3 ± 10.28 |
|  |  | F (3) | 4.0 ± 0.58 | 349.0 ± 18.34 | 564.3 ± 92.08 | 1.25 ± 0.07 | 1.27 ± 0.09 | 120.0 ± 11.55 |
