# Supplementary material for: Temporal analysis of water chemistry and smallmouth bass (Micropterus dolomieu) health at two sites with divergent land use in the Susquehanna River watershed, Pennsylvania, USA
Source: Environ Monit Assess. 2024 Sep 11;196(10):922. doi: 10.1007/s10661-024-13049-4 (PMC11390901; doi:10.1007/s10661-024-13049-4)
Supplement: Supplementary file 2 — Supplementary file2 (DOCX 19 KB) [file 10661_2024_13049_MOESM2_ESM.docx]

|  | Sampling Date | Sex | Liver Para/mm^2^ | Spleen Para/mm^2^ | Liver MA/mm^2^ | Spleen MA/mm^2^ |
| --- | --- | --- | --- | --- | --- | --- |
| Pine | 4/15/2015 | M (17) | 23.2 ± 3.13 | 7.6 ± 1.60 | 124.9 ± 17.61 | 546.3 ± 59.32 |
|  |  | F (2) | 12.6 ± 8.39 | 1.6 ± 1.58 | 74.7 ± 19.66 | 585.8 ± 192.66 |
|  | 5/2/2016 | M (13) | 10.0 ± 1.90 | 3.1 ± 0.80 | 90.1 ± 18.37 | 402.4 ± 56.19 |
|  |  | F (7) | 2.7 ± 0.75 | 1.4 ± 0.64 | 51.7 ± 12.48 | 423.2 ± 89.35 |
|  | 10/11/2016 | M (7) | 25.8 ± 5.67 | 10.3 ± 3.74 | 53.9 ± 6.05 | 340.4 ± 68.50 |
|  |  | F (9) | 47.1 ± 16.89 | 31.1 ± 10.72 | 63.8 ± 6.74 | 399.3 ± 75.25 |
|  | 4/26/2017 | M (8) | 12.6 ± 3.41 | 6.3 ± 1.88 | 88.5 ± 25.73 | 387.3 ± 65.32 |
|  |  | F (12) | 20.8 ± 11.71 | 13.9 ± 6.69 | 43.6 ± 7.67 | 333.5 ± 43.70 |
|  | 10/3/2017 | M (9) | 22.8 ± 7.15 | 15.4 ± 4.82 | 39.3 ± 9.08 | 167.8 ± 33.85 |
|  |  | F (11) | 27.8 ± 4.84 | 12.3 ± 3.76 | 49.3 ± 5.52 | 203.0 ± 21.04 |
|  | 5/14/2018 | M (13) | 19.0 ± 4.41 | 16.0 ± 2.93 | 48.4 ± 4.44 | 395.0 ± 56.38 |
|  |  | F (6) | 24.8 ± 4.58 | 21.0 ± 5.89 | 35.4 ± 5.65 | 243.8 ± 39.27 |
|  | 10/10/2018 | M (4) | 15.2 ± 8.23 | 5.5 ± 3.24 | 19.7 ± 7.53 | 249.7 ± 143.98 |
|  |  | F (14) | 16.9 ± 2.63 | 5.3 ± 2.00 | 50.0 ± 7.22 | 384.1 ± 80.10 |
|  | 5/29/2019 | M (16) | 15.2 ± 3.28 | 7.5 ± 2.28 | 24.6 ± 3.12 | 226.6 ± 33.34 |
|  |  | F (2) | 4.2 ± 0.00 | 7.9 ± 4.72 | 27.5 ± 27.52 | 479.7 ± 188.73 |
| WBM | 5/7/2015 | M (7) | 3.0 ± 1.01 | 1.4 ± 0.64 | 62.9 ± 10.30 | 325.8 ± 86.38 |
|  |  | F (10) | 1.9 ± 0.79 | 3.8 ± 2.09 | 55.8 ± 9.41 | 432.5 ± 71.42 |
|  | 4/25/2016 | M (8) | 4.2 ± 1.94 | 2.8 ± 0.95 | 99.3 ± 16.94 | 607.0 ± 86.35 |
|  |  | F (12) | 1.6 ± 0.86 | 0.7 ± 0.54 | 73.4 ± 8.68 | 646.6 ± 64.89 |
|  | 10/11/2016 | M (11) | 15.6 ± 3.65 | 14.3 ± 5.57 | 21.4 ± 3.98 | 85.1 ± 17.74 |
|  |  | F (6) | 8.0 ± 4.94 | 5.2 ± 1.93 | 15.7 ± 2.87 | 104.8 ± 28.62 |
|  | 4/20/2017 | M (10) | 2.5 ± 0.93 | 0.7 ± 0.70 | 54.3 ± 9.84 | 270.0 ± 53.53 |
|  |  | F (10) | 0.8 ±0.46 | 0.00 | 52.7 ± 10.62 | 362.5 ± 67.87 |
|  | 10/2/2017 | M (12) | 12.6 ± 2.22 | 10.7 ± 4.18 | 97.0 ± 22.60 | 380.7 ± 91.03 |
|  |  | F (8) | 18.6 ± 5.47 | 14.9 ± 7.20 | 54.1 ± 6.03 | 217.2 ± 57.84 |
|  | 5/7/2018 | M (9) | 7.5 ± 2.45 | 1.7 ± 0.76 | 60.3 ± 10.97 | 222.8 ± 44.85 |
|  |  | F (10) | 1.5 ± 0.55 | 1.3 ± 0.70 | 43.2 ± 5.53 | 329.5 ± 39.45 |
|  | 10/2/2018 | M (9) | 10.5 ± 5.41 | 4.2 ± 1.74 | 94.4 ± 19.31 | 423.8 ± 76.65 |
|  |  | F (11) | 10.5 ± 3.24 | 4.0 ± 1.85 | 95.1 ± 18.38 | 355.3 ± 79.74 |
|  | 5/28/2019 | M (16) | 8.7 ± 2.07 | 3.9 ± 1.33 | 44.7 ± 6.80 | 323.4 ± 38.40 |
|  |  | F (3) | 7.7 ± 3.70 | 6.3 ± 3.14 | 49.8 ± 9.45 | 361.7 ± 170.90 |
