# Supplementary material for: Temporal analysis of water chemistry and smallmouth bass (Micropterus dolomieu) health at two sites with divergent land use in the Susquehanna River watershed, Pennsylvania, USA
Source: Environ Monit Assess. 2024 Sep 11;196(10):922. doi: 10.1007/s10661-024-13049-4 (PMC11390901; doi:10.1007/s10661-024-13049-4)
Supplement: Supplementary file 3 — Supplementary file3 (DOCX 15 KB) [file 10661_2024_13049_MOESM3_ESM.docx]

|  | **WBM HSI** | | | **WBM HAI** | | |
| --- | --- | --- | --- | --- | --- | --- |
| *Predictors* | *Estimates* | *CI* | *p* | *Estimates* | *CI* | *p* |
| (Intercept; Season (Fall), Sex (F)) | 260.130 | 169.372 – 351.889 | **<0.001** | -645.178 | -12,702.669  – 11,412.312 | 0.916 |
| *egfr* | -4.203e-6 | -2.611e-5 – 1.770e-5 | 0.704 |  |  |  |
| *hprt1* | 1.483e-3 | 8.824e-4 – 2.084e-3 | **<0.001** |  |  |  |
| Year | -0.129 | -0.174 – -0.083 | **<0.001** | 0.329 | -5.652 – 6.310 | 0.913 |
| Season (Spring) | 0.210 | 0.104 – 0.314 | **<0.001** | -9.107 | -26.609 – 8.394 | 0.305 |
| Age | 0.020 | -0.013 – 0.052 | 0.227 | 6.412 | 2.420 – 10.404 | **0.002** |
| Sex (M) | -0.253 | -0.363 – -0.143 | **<0.001** | -4.199 | -20.734 – 12.337 | 0.616 |
| *apa1* |  |  |  | 1.193e-4 | 4.996e-5 – 1.886e-4 | **0.001** |
| *tf* |  |  |  | 1.860e-4 | 7.262e-5 – 2.994e-4 | **0.002** |
| *c3* |  |  |  | -6.360e-4 | -0.001 – -1.021e-5 | **0.046** |
| Observations | 129 | | | 129 | | |
| R^2^ | 0.467 | | | 0.298 / 0.257 | | |
