# Supplementary material for: Temporal analysis of water chemistry and smallmouth bass (Micropterus dolomieu) health at two sites with divergent land use in the Susquehanna River watershed, Pennsylvania, USA
Source: Environ Monit Assess. 2024 Sep 11;196(10):922. doi: 10.1007/s10661-024-13049-4 (PMC11390901; doi:10.1007/s10661-024-13049-4)
Supplement: Supplementary file 4 — Supplementary file4 (DOCX 15 KB) [file 10661_2024_13049_MOESM4_ESM.docx]

|  | **Pine HSI** | | | **Pine HAI** | | |
| --- | --- | --- | --- | --- | --- | --- |
| *Predictors* | *Estimates* | *CI* | *p* | *Estimates* | *CI* | *p* |
| (Intercept; Season (Fall), Sex (F)) | -191.323 | -294.430 –  -88.217 | **<0.001** | -26,615.967 | -36,344.949 –  -16,886.984 | **<0.001** |
| *gsr* | 0.003 | 0.002 – 0.005 | **<0.001** |  |  |  |
| Season (Spring) | 0.287 | 0.134 – 0.440 | **<0.001** | -11.278 | -23.902 – 1.346 | 0.079 |
| *ho1b* | -6.302e-4 | -0.004 – 0.002 | 0.689 |  |  |  |
| Year | 0.095 | 0.044 – 0.146 | **<0.001** | 13.208 | 8.387 – 18.030 | **<0.001** |
| Age | 0.005 | -0.026 – 0.036 | 0.762 | 5.915 | 3.120 – 8.709 | **<0.001** |
| Sex (M) | -0.341 | -0.482 – -0.199 | **<0.001** | 1.178 | -10.182 – 12.538 | 0.838 |
| *gst* |  |  |  | 0.003 | -0.002 – 0.008 | 0.239 |
| *igf1* |  |  |  | 0.025 | -0.007 – 0.057 | 0.128 |
| Observations | 127 | | | 127 | | |
| R^2^ / R^2^ adjusted | 0.533 / 0.509 | | | 0.292 / 0.256 | | |
