# Supplementary material for: Temporal analysis of water chemistry and smallmouth bass (Micropterus dolomieu) health at two sites with divergent land use in the Susquehanna River watershed, Pennsylvania, USA
Source: Environ Monit Assess. 2024 Sep 11;196(10):922. doi: 10.1007/s10661-024-13049-4 (PMC11390901; doi:10.1007/s10661-024-13049-4)
Supplement: Supplementary file 5 — Supplementary file5 (DOCX 15 KB) [file 10661_2024_13049_MOESM5_ESM.docx]

|  | **WBM Liver Parasite Density** | | | **WBM Spleen Parasite Density** | | |
| --- | --- | --- | --- | --- | --- | --- |
| *Predictors* | *Estimates* | *CI* | *p* | *Estimates* | *CI* | *p* |
| (Intercept; Season (Fall), Sex (F)) | -1,227.044 | -4,476.306 –  2,022.218 | 0.456 | 688.830 | -2,614.781 –  3,992.440 | 0.680 |
| *apa1* | 7.000e-8 | -1.860e-5 –  1.875e-5 | 0.994 | -3.570e-6 | -2.255e-5 –  1.542e-5 | 0.711 |
| *c3* | -8.390e-6 | -1.770e-4 –  1.603e-4 | 0.922 | 1.137e-4 | -5.780e-5 –  2.851e-4 | 0.192 |
| *tf* | 1.345e-5 | -1.711e-5 –  4.401e-5 | 0.385 | 1.310e-6 | -2.976e-5 –  3.238e-5 | 0.933 |
| Season (Spring) | -8.589 | -13.306 –  -3.837 | **<0.001** | -4.389 | -9.184 – 0.406 | 0.072 |
| Year | 0.615 | -0.997 – 2.227 | 0.451 | -0.336 | -1.975 – 1.302 | 0.685 |
| Sex (M) | 1.351 | -3.105 – 5.807 | 0.549 | -0.816 | -5.347 – 3.715 | 0.722 |
| Age | -0.671 | -1.747 – 0.405 | 0.220 | -1.402 | -2.495 – -0.308 | **0.012** |
| Observations | 129 | | | 129 | | |
| R^2^ / R^2^ adjusted | 0.230 / 0.185 | | | 0.208 / 0.163 | | |
