# Supplementary material for: Temporal analysis of water chemistry and smallmouth bass (Micropterus dolomieu) health at two sites with divergent land use in the Susquehanna River watershed, Pennsylvania, USA
Source: Environ Monit Assess. 2024 Sep 11;196(10):922. doi: 10.1007/s10661-024-13049-4 (PMC11390901; doi:10.1007/s10661-024-13049-4)
Supplement: Supplementary file 6 — Supplementary file6 (DOCX 15 KB) [file 10661_2024_13049_MOESM6_ESM.docx]

|  | **WBM Liver MA Density** | | | **WBM Spleen MA Density** | | |
| --- | --- | --- | --- | --- | --- | --- |
| *Predictors* | *Estimates* | *CI* | *p* | *Estimates* | *CI* | *p* |
| (Intercept; Season (Fall), Sex (F)) | 5,072.454 | -3,501.260 –  13,646.168 | 0.244 | 43,078.356 | -156,861.973 –  102,018.664 | 0.150 |
| *glk* | -0.009 | -0.019 – 0.002 | 0.097 |  |  |  |
| *mt* | 0.005 | -1.799e-5 – 0.011 | 0.051 |  |  |  |
| Season (Spring) | -21.538 | -32.037 – -11.039 | **<0.001** | 25.439 | -60.113 – 110.990 | 0.557 |
| Year | -2.523 | -6.776 – 1.729 | 0.242 | -21.345 | -50.581 – 7.892 | 0.151 |
| Age | 20.587 | 17.578 – 23.596 | **<0.001** | 78.503 | 58.988 – 98.018 | **<0.001** |
| Sex (M) | 7.478 | -2.191 – 17.146 | 0.128 | -0.443 | -81.275 – 80.389 | 0.991 |
| *apa1* |  |  |  | -2.974e-4 | -6.361e-4 – 4.140e-5 | 0.085 |
| *c3* |  |  |  | 2.698e-5 | -0.003 – 0.003 | 0.986 |
| *tf* |  |  |  | 2.487e-4 | -3.057e-4 – 8.030e-4 | 0.376 |
| Observations | 129 | | | 129 | | |
| R^2^ / R^2^ adjusted | 0.656 / 0.639 | | | 0.442 / 0.409 | | |
