# Supplementary material for: Temporal analysis of water chemistry and smallmouth bass (Micropterus dolomieu) health at two sites with divergent land use in the Susquehanna River watershed, Pennsylvania, USA
Source: Environ Monit Assess. 2024 Sep 11;196(10):922. doi: 10.1007/s10661-024-13049-4 (PMC11390901; doi:10.1007/s10661-024-13049-4)
Supplement: Supplementary file 7 — Supplementary file7 (DOCX 14 KB) [file 10661_2024_13049_MOESM7_ESM.docx]

|  | **Pine Liver Parasite Density** | | | **Pine Spleen Parasite** | | |
| --- | --- | --- | --- | --- | --- | --- |
| *Predictors* | *Estimates* | *CI* | *p* | *Estimates* | *CI* | *p* |
| (Intercept; Season (Fall), Sex (F)) | 7,130.753 | 40.169 – 14,221.339 | **0.049** | 3,055.654 | -1,742.533 –  7,853.841 | 0.210 |
| Season (Spring) | -4.818 | -14.149 – 4.513 | 0.309 | 0.944 | -5.370 – 7.258 | 0.768 |
| Year | -3.521 | -7.035 – -0.007 | 0.050 | -1.505 | -3.883 – 0.872 | 0.212 |
| Age | -0.321 | -2.407 – 1.764 | 0.761 | -1.106 | -2.518 – 0.305 | 0.123 |
| Sex (M) | -4.075 | -12.463 – 4.313 | 0.338 | -5.085 | -10.761 –  0.591 | 0.079 |
| Observations | 127 | | | 127 | | |
| R^2^ / R^2^ adjusted | 0.066 / 0.035 | | | 0.053 / 0.022 | | |
