# Supplementary material for: Temporal analysis of water chemistry and smallmouth bass (Micropterus dolomieu) health at two sites with divergent land use in the Susquehanna River watershed, Pennsylvania, USA
Source: Environ Monit Assess. 2024 Sep 11;196(10):922. doi: 10.1007/s10661-024-13049-4 (PMC11390901; doi:10.1007/s10661-024-13049-4)
Supplement: Supplementary file 8 — Supplementary file8 (DOCX 15 KB) [file 10661_2024_13049_MOESM8_ESM.docx]

|  | **Pine Liver MA Density** | | | **Pine Spleen MA Density** | | |
| --- | --- | --- | --- | --- | --- | --- |
| *Predictors* | *Estimates* | *CI* | *p* | *Estimates* | *CI* | *p* |
| (Intercept; Season (Fall), Sex (F)) | 17,527.31 | 7,511.144 – 27,543.475 | **0.001** | 45,651.730 | -23,662.207 –  114,965.667 | 0.195 |
| *cyp3a* | -5.493e-4 | -0.013 – 0.012 | 0.929 | 0.064 | -0.020 – 0.149 | 0.134 |
| *efgr* | -0.018 | -0.210 – 0.174 | 0.853 | 0.451 | -0.877 – 1.778 | 0.503 |
| *hep2* | 0.002 | -0.018 – 0.022 | 0.847 | -0.049 | -0.186 – 0.088 | 0.482 |
| *mt* | 0.003 | -2.547e-4 – 0.006 | 0.072 | 0.010 | -0.011 – 0.031 | 0.353 |
| Season (Spring) | -9.696 | -22.391 – 3.000 | 0.133 | -1.525 | -89.376 –  86.326 | 0.973 |
| Year | -8.685 | -13.650 – -3.721 | **0.001** | -22.583 | -56.940 – 11.775 | 0.196 |
| Age | 11.106 | 8.232 – 13.980 | **<0.001** | 36.667 | 16.780 – 56.555 | **<0.001** |
| Sex (M) | 4.829 | -7.640 – 17.298 | 0.445 | -14.151 | -100.438 – 72.136 | 0.746 |
| Observations | 127 | | | 127 | | |
| R^2^ / R^2^ adjusted | 0.598 / 0.570 | | | 0.257 / 0.207 | | |
